# Methicillin-resistant *Staphylococcus aureus* in Veterinary Doctors and Students, the Netherlands

**DOI:** 10.3201/eid1212.060355

**Published:** 2006-12

**Authors:** Mireille Wulf, Arie van Nes, Andrea Eikelenboom-Boskamp, Janneke de Vries, Willem Melchers, Corné Klaassen, Andreas Voss

**Affiliations:** * Radboud University Nijmegen Medical Centre, Nijmegen, the Netherlands;; † Utrecht University, Utrecht, the Netherlands;; ‡ Canisius Wilhelmina Hospital, Nijmegen, the Netherlands

**Keywords:** Staphylococcus aureus, methicillin resistance, veterinarian, domestic animals, carriers, research

## Abstract

The prevalence of methicillin-resistant *Staphylococcus aureus* (MRSA) in the Netherlands, at 1.0%, is among the lowest in Europe. In 2004, a relationship between pig farming and a high risk for MRSA carriage was found. To investigate if those in professional contact with livestock are at higher risk for MRSA carriage, we screened 80 veterinary students and 99 veterinarians and questioned them about animal contacts and known MRSA risk factors. Of these, 27 students who did not have livestock contact were excluded from further analysis. We found 7 carriers of MRSA, a prevalence of 4.6%, which is similar to that found in patients who had previously been treated at foreign hospitals. A correlation of MRSA carriage with a specific animal group could not be established. To preserve the low prevalence of MRSA in the Netherlands, persons involved in the care of livestock should be isolated and screened on admission to the hospital.

In the Netherlands, the prevalence of methicillin-resistant Staphylococcus aureus (MRSA) in clinical isolates of S. aureus has been <1% during the past decade (*1**,**2*) and, at 1.0%, remains one of the lowest in Europe (*3*). This low prevalence is best explained by the national "search and destroy" policy, which demands isolation and screening of patients at risk for MRSA carriage on admission to healthcare facilities. So far, the patients at risk have mainly been persons who had previously been admitted to or treated in foreign hospitals.

In 2004, three patients in our hospital who had no relation to foreign hospitals or exposure to other known sources of MRSA were unexpectedly found to carry MRSA. The patients were a pig farmer, a pig farmer's child, and a veterinarian's child. A subsequent screening of local pig farmers showed MRSA prevalence of >20%, which suggested that contact with pigs, at least in that region of the Netherlands, posed a substantial risk of acquiring MRSA (*4*). If that hypothesis were true, isolation on admission and screening of pig farmers and their family members for MRSA would be indicated. To further investigate if those in professional contact with livestock are at higher risk for MRSA carriage, we screened a selection of veterinary doctors and students.

## Materials and Methods

We screened 80 veterinary students in the last phases of their education and 99 veterinarians attending a conference on livestock. Cultures were taken from both anterior nares and throat. All participants were asked to fill in a questionnaire about the type of animal contacts and possible exposure to known MRSA risk factors.

We incubated all cultures in a salt-enriched nutrient broth and after 24 hours subcultured them on blood agar plates and MRSA-ID agars (bioMerieux, La Balme Les Grottes, France). Colony morphology and latex agglutination test (Staphaurex, Remel, Lenexa, KS, USA) initially identified staphylococci; cefoxitine-disc diffusion determined methicillin resistance, according to Clinical and Laboratory Standards Institute criteria (*5*). All cefoxitine-resistant isolates underwent further identification and susceptibility testing to cefoxitine, gentamicin, vancomycin, teicoplanin, clindamycin, erythromycin, rifampicin, ciprofloxacin, cotrimoxazol, and tetracycline, using the Phoenix Automated Microbiology System (Becton Dickinson, Franklin Lakes, NJ, USA). We also performed mecA gene PCR, typing by pulsed-field gel electrophoresis (PFGE) with SmaI (the standard method), and spa-typing on all cefoxitine-resistant strains.

## Results

The main characteristics of the veterinary doctors and students are listed in Table 1. Among the 179 persons tested, 7 (3.9%) MRSA carriers were found: 2 students and 5 veterinarians (Table 2). MRSA carriage varied depending on whether or not study participants had contact with livestock. MRSA carriage was 4.6% among 152 students and doctors in contact with livestock and 0% among 27 students who reported no contact with livestock. All MRSA carriers in this study had recent or regular contact with pigs and cows; only 3 veterinarians reported regular contact with sheep. Because all carriers reported contact with cows and pigs, no relative risk could be calculated (Table 3). In each group, 1 person indicated a known risk factor for MRSA carriage (1 had been admitted to a foreign hospital; 1 had an MRSA-positive family member), but both tested MRSA negative.

**Table 1:** Main characteristics of veterinary students and veterinarians, the Netherlands

Characteristics	Veterinary students, n = 80, no. (%)	Veterinarians, n = 99, no. (%)
Mean age (range), y	26 (23–41)	43 (27–60)
Male	24 (30)	83 (83)
Professional contact limited to livestock	49 (63)	72 (73)
Professional contact limited to companion animals	27 (32)	0
Professional contact with livestock and companion animals	4 (5)	27 (27)
Contact with cows	48 (60)*	83 (83)†
Contact with pigs	37 (47)	72 (72)†
Contact with sheep	Not known	36 (36)†
Contact with pets at home	52 (65)	81 (81)
Risk factors for MRSA carriage‡	1 (1.2)	1 (1)

*Regular contact in past 3 months. †Regular part of practice and/or regular contact in the past 6 months. ‡MRSA, methicillin-resistant *Staphylococcus aureus*.

**Table 2:** Characteristics and type of animal contact of MRSA carriers, the Netherlands*

Case	Sex	Profession	Pigs	Cows	Horses	Sheep	Companion animals
1	F	Student	X	X		?	
2	F	Student	X	X	X	?	
3	M	Veterinarian	X	X	X	X	X
4	M	Veterinarian	X	X	X		X
5	M	Veterinarian	X	X		X	
6	M	Veterinarian	X	X		X	
7	M	Veterinarian	X	X			
*MRSA, methicillin-resistant *Staphylococcus aureus*.							

**Table 3:** Estimates of relative risk for exposure to types of animals for veterinary students and veterinarians, the Netherlands

Type of animal	Relative risk	95% Confidence interval
Pigs*	9.0	0.52–154
Cows*	5.3	0.31–90
Sheep†	4.35	0.52–40
Companion animals	0.86	0.17–4.2
Horses	0.72	0.14–3.6

*The number of carriers without exposure in this group was 0; estimate of relative risk was made by adding 0.5 to all groups. †Data on veterinarians only.

In addition to 7 MRSA isolates, S. sciuri was isolated from 1 veterinarian. This strain showed green colonies on the ID-MRSA plates and was Staphaurex positive (http://www.dcss.cs.amedd.army.mil/field/FLIP30/documents/pdfs/staphaurex_insert.pdf), which caused the risk to be wrongly identified as MRSA.

All cefoxitine-resistant isolates were susceptible to vancomycin, teicoplanin, rifampicin, and ciprofloxacin, but all were resistant to tetracycline. All MRSA strains and the S. sciuri were mecA positive and were resistant to digestion with restriction endonuclease SmaI when typing by PFGE was attempted, similar to the strains described by Voss et al. (*4*). Overall, 3 different MRSA types were identified by spa typing; 2 students and 1 veterinarian carried spa-type t011, 3 veterinarians carried spa-type t108, and 1 veterinarian carried spa-type t034. In contrast to the study of Voss et al., t108 was not a dominant spa-type.

## Discussion

MRSA has been found in various animals, such as horses (*6*) and livestock (*7*), including pigs (*4**,**8*). So far, only 1 study has indicated transmission from livestock to caretakers (*4*). The extent of this transmission and its clinical significance remain unknown, also undetermined is whether persons in professions other than farming are at increased risk of becoming MRSA carriers. The overall MRSA prevalence in veterinary students and doctors involved in farm animal health in the Netherlands was about 160× higher than that among patients at hospital admissions (4.6% vs. 0.03%) (*9*); this prevalence falls within the range of that found in patients from foreign hospitals (3.5%–5%) (*10*). At least with regard to the search and destroy policy in the Netherlands, veterinarians and veterinary students who come in contact with the healthcare system may therefore qualify as patients at high risk, warranting screening and isolation on admission to hospitals.

The high frequency of MRSA carriage among veterinary doctors and students is unexpected. While protective coveralls and boots are routinely used during veterinary contact with livestock, protective masks are not. Because S. aureus colonization and transmission occur mainly through contact from the hands to the anterior nares, the standard measures are probably insufficient to prevent MRSA colonization. Therefore, masks and gloves could be considered as additional protective measures.

Although low in comparison with several other countries, the quantity and intensity of antimicrobial use in livestock has increased in the Netherlands (*11*). Data from 1997 to 2004 show that the main antimicrobial classes used in livestock are tetracycline and trimethoprim sulfonamide combinations. All the MRSA strains in this study, and all the strains found by Voss et al., were resistant to tetracycline.

We conclude that veterinary doctors and students caring for livestock have a high risk of being colonized by MRSA. The percentage of MRSA carriage in the doctors and students surveyed is such that, to preserve the low prevalence of MRSA in the Netherlands, all persons involved in the care of livestock should be isolated and screened on admission to the hospital, according to national policy. Further investigation is needed to determine the exact source of MRSA in livestock and the effect of risk factors such as the use of antimicrobial agents on MRSA carriage in livestock. This type of research should be conducted in other countries to find out if this phenomenon is limited to the Netherlands or is international.

## References

[1] Tiemersma EW, Bronzwaer SL, Degener JE, Lyytikäinen O, Schrijnemakers P, Bruinsma N, Methicillin-resistant *Staphylococcus aureus* in Europe, 1999–2002. Emerg Infect Dis. 2004;10:1627–34. 15498166 10.3201/eid1009.040069 PMC3320277

[2] Voss A, Milatovic D, Wallrauch-Schwarz C, Rosdahl VT, Braveny I. Methicillin-resistant *Staphylococcus aureus* in Europe. Eur J Clin Microbiol Infect Dis. 1994;13:50–5. 10.1007/BF02026127 8168564

[3] European Antimicrobal Resistance Surveillance System (EARSS). EARSS annual report 2004. [cited 2006 Oct 4]. Available from http://www.rivm.nl/earss/Images/EARSS%20annual%20report%202004%20webversie_tcm61-25345.pdf

[4] Voss A, Loeffen F, Bakker J, Wulf M, Klaassen C. Methicillin-resistant *Staphylococcus aureus* in pig farming. Emerg Infect Dis. 2005;11:1965–6. 16485492 10.3201/eid1112.050428 PMC3367632

[5] Clinical and Laboratory Standards Institute (CLSI). Performance standards for antimicrobial susceptibility testing; 15th informational supplement. CLSI/NCCLS M100-S15. Wayne (PA): The Institute; 2005.

[6] Weese JS, Archambault M, Willey BM, Dick H, Hearn P, Kreiswirth BN, Methicillin-resistant *Staphylococcus aureus* in horses and horse personnel, 2000–2002. Emerg Infect Dis. 2005;11:430–5. 15757559 10.3201/eid1103.040481 PMC3298236

[7] Lee JH. Methicillin (oxacillin)-resistant *Staphylococcus aureus* strains isolated from major food animals and their potential transmission to humans. Appl Environ Microbiol. 2003;69:6489–94. 10.1128/AEM.69.11.6489-6494.2003 14602604 PMC262320

[8] Armand-Lefevre L, Ruimy R, Andremont A. Clonal comparison of *Staphylococcus aureus* isolates from healthy pig farmers, human controls, and pigs. Emerg Infect Dis. 2005;11:711–4. 15890125 10.3201/eid1105.040866 PMC3320358

[9] Wertheim HF, Vos MC, Boelens HA, Voss A, Vandenbroucke-Grauls CM, Meester MH, Low prevalence of methicillin resistant *Staphylococcus aureus* (MRSA) at hospital admission in the Netherlands: the value of search and destroy and restrictive antibiotic use. J Hosp Infect. 2004;56:321–5. 10.1016/j.jhin.2004.01.026 15066745

[10] Kaiser AM, Schultsz C, Kruithof GJ, Debets-Ossenkopp YJ, Vandenbroucke-Grauls CM. Carriage of resistant micro-organisms in repatriates from foreign hospitals to the Netherlands. Clin Microbiol Infect. 2004;110:972–9. 10.1111/j.1469-0691.2004.01000.x 15521999

[11] Mevius DJ, Pellicaan C, van Pelt W. MARAN-2004: monitoring of antimicrobial resistance and antibiotic usage in animals in the Netherlands in 2004. [cited 2006 Oct 6]. Available from http://www.cidc-lelystad.wur.nl/NR/rdonlyres/7F79ACE6-0FD2-41AB-81B2-BB17FA89603C/11382/MARAN2004web1.pdf

